# High Cholesterol Deteriorates Bone Health: New Insights into Molecular Mechanisms

**DOI:** 10.3389/fendo.2015.00165

**Published:** 2015-10-23

**Authors:** Chandi C. Mandal

**Affiliations:** ^1^Department of Biochemistry, School of Life Sciences, Central University of Rajasthan, Rajasthan, India

**Keywords:** osteoporosis, bone mineral density, bone remodeling, bone metastasis, osteoblast, osteoclast, cholesterol, statins

## Abstract

Many epidemiological studies show a positive connection between cardiovascular diseases and risk of osteoporosis, suggesting a role of hyperlipidemia and/or hypercholesterolemia in regulating osteoporosis. The majority of the studies indicated a correlation between high cholesterol and high LDL-cholesterol level with low bone mineral density, a strong predictor of osteoporosis. Similarly, bone metastasis is a serious complication of cancer for patients. Several epidemiological and basic studies have established that high cholesterol is associated with increased cancer risk. Moreover, osteoporotic bone environment predisposes the cancer cells for metastatic growth in the bone microenvironment. This review focuses on how cholesterol and cholesterol-lowering drugs (statins) regulate the functions of bone residential osteoblast and osteoclast cells to augment or to prevent bone deterioration. Moreover, this study provides an insight into molecular mechanisms of cholesterol-mediated bone deterioration. It also proposes a potential mechanism by which cellular cholesterol boosts cancer-induced bone metastasis.

## Introduction

As the life expectancy increases, the risk of developing various diseases, such as cardiovascular diseases (CVD), osteoporosis, and cancer, increases with aging. Osteoporosis is a major health concern, especially for menopausal women, and it accounts for a significant economic burden worldwide. Epidemiological studies have linked an association of CVD with reduced bone mineral density (BMD) and with increased bone fracture (1–3). Low BMD is a strong predictor for osteoporosis-associated fracture risk.

Many, but not all, studies have suggested a positive link between dyslipidemia [such as high serum total cholesterol (TC) and high low-density lipoprotein cholesterol (LDL-C)] and low BMD, especially for postmenopausal women (4–8). Moreover, a reduction of BMD was reported, when rodents were kept on high fat diet (9). Various clinical and animal studies have evidenced that cholesterol-lowering statin drugs, reduce osteoporosis-associated fracture risk (10–12). Similarly, a relationship between cancer risk and hypercholesterolemia was found from many epidemiological and basic studies (13–17). In our studies, we have demonstrated the active role of statins in osteoblast differentiation, which supports bone formation, but inhibited osteolytic metastasis of breast cancer (18, 19).

In this review, we have attempted to address the effect of cholesterol and statins on the activities of osteoblast and osteoclast cells, and overall bone health. This report also highlights the underlying molecular mechanisms of high cholesterol-mediated bone deterioration.

## Involvement of Various Cell Types in Bone Remodeling

Bone is an active and highly dynamic tissue, which is continuously remodeled by a balanced and coordinated action of bone-forming osteoblast and bone-resorbing osteoclast cells. In the osteoporotic condition, the rate of bone resorption exceeds that of bone formation either due to enhancement of osteoclast activity or impairment of osteoblast differentiation. Bone residential osteoblast, osteoclast, and osteocyte cells primarily regulate this physiological process. Mature osteoblast cells release several bone matrix proteins, such as collagen 1, osteocalcin (OCN), bone sialo protein (BSP), and osteopontin (OPN), to the extracellular environment. These components form osteoid (soft bone) (20). Osteoblasts also facilitate the formation of hydroxyapatite (HA; CaPO4) crystal inside the matrix vesicles (MVs). These MVs move to the extracellular environment, and unload the crystal molecules on the top of osteoid, which provides the cementing material, to make the matrix hard (21). In contrast to osteoblasts, multinucleated giant osteoclast cells release H^+^ ion to dissolve this cementing material, and secrete various proteolytic enzymes, such as tartrate resistant acid phosphatase (TRAP), collagenolytic cathepsin K, and matrix metalloproteinases (MMPs), to break down the bone matrix.

Osteoblasts/osteoblast lineage cells also produce osteoclastogenic factors, such as colony stimulating factor-1 (CSF-1), receptor activator of NFκB ligand (RANKL), and osteoprotegerin OPG (20). These CSF-1 and RANKL bind to their receptor CSF-1R and RANK present in osteoclast precursor monocyte/macrophage cells. These ligand–receptor interactions transduce different intracellular signaling cascades inside osteoclast cells, and subsequently promote survival, proliferation, and differentiation of osteoclast cells, and eventually activate osteoclast activity. Osteocytes are terminally differentiated osteoblast cells, and they are embedded into the bone matrix. Accumulating evidence documents that dendritic osteocytes interconnect with other bone cells through gap junction, and also sense the mechanical stress, minor crack, and changes in hormonal milieu of the bone (22, 23). Moreover, osteocyte cell-derived soluble factors, such as sclerostin, fibroblast growth factor (FGF) 23, OPG, and RANKL, regulate bone function, bone remodeling, and bone metabolism (24).

**Figure 1:**
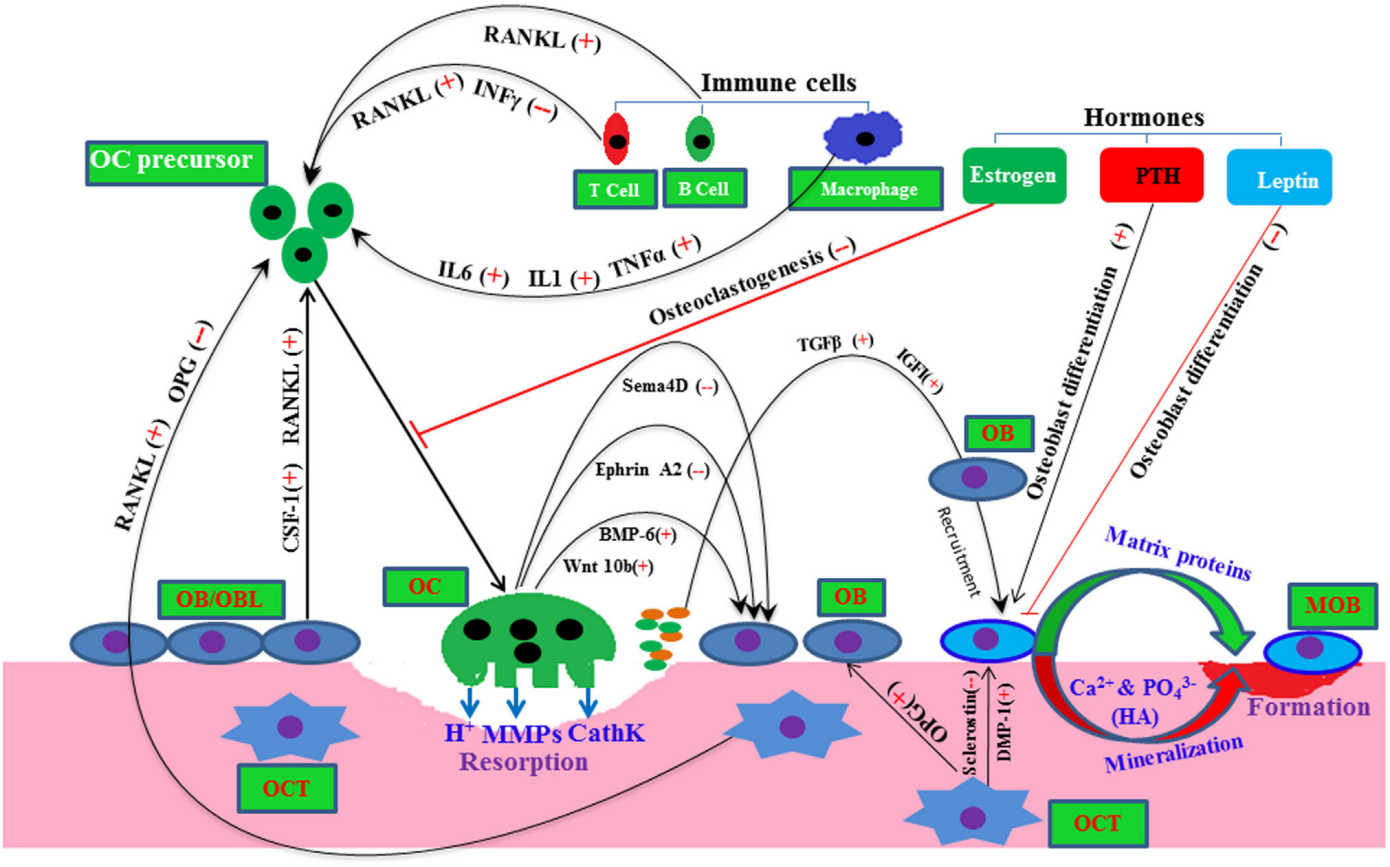
**A molecular view of bone remodeling with a complex interaction among bone resident cells and other cells**. Here, plus and minus signs depict positive and negative regulator, respectively, line arrow head and blunt ended line depict increased and decreased function, respectively. Abbreviations: OC, osteoclast; OB, osteoblast; OBL, osteoblast lineage; MOB, mature osteoblast; OCT, osteocyte; HA, hydroxyapatite; Sema4D, semasporin 4D; CathK, cathepsin K; PTH, parathyroid hormone.

Recent studies have introduced novel molecular mechanisms by which osteoclast-derived factors, such as Wnt10, BMP-6, EphrinA2, and Semaphorin (SemaD4), regulate osteoblast activity (24, 25) (Figure 1). During the course of osteoclastic bone resorption, TGFβ and IGF-I are released in a large amount from the embedded bone matrix, and these growth factors may promote the recruitment and activation of osteoblasts in the bone multicellular units (26). Bone morphogenetic proteins (BMPs) are the most potent osteoinducers, which play a pivotal role in bone remodeling by regulating both osteoblast and osteoclast activity (27–29).

Other cell types, such as immune T cells, B cells, and stromal cells, also influence this bone remodeling process by supplying osteoclastogenic RANKL and anti-osteoclastogenic interferon gamma (INFγ) to the osteoclast precursor cells (Figure 1) (30, 31). In pathophysiology condition-like arthritis, T and B cells both may increase osteoclastogenesis presumably by supplying RANKL to osteoclast cells (30–32). Various hormones, such as leptin, parathyroid hormone (PTH), and sex hormones, have been shown to regulate osteoblastic/osteoclastic function (23, 26, 33) (Figure 1). Thus, systemic and local environments manipulate the bone function, bone metabolism, and bone remodeling (34).

## Cholesterol-Lowering Statin Drugs and Health Benefit

Statins are the most prescribed cholesterol-lowering drugs. Different members of statins, such as simvastatin, lovastatin, atorvastatin, pravastatin, and mevastatin, are therapeutically used for the treatment of CVD-like atherosclerosis. In all, statin treatment lowers 25–35% of serum LDL-cholesterol and reduces 25–30% frequency of heart attack (35). Statins competitively inhibit the activity of 3-hydroxy-3-methylglutaryl-coenzyme A (HMGCOA) reductase, a rate limiting enzyme of the cholesterol biosynthesis pathway in a reversible fashion (36, 37). *In vitro*, *in vivo*, and clinical studies show very promising results in reducing osteoporosis and cancers for statin users (10–12, 38). However, many studies have reported conflicting findings in cases of type 2 diabetes for statin users (39, 40).

## Cholesterol and Osteoblasts

Research studies were carried out in cells, animal models, and clinical samples to evaluate the influence of serum cholesterol and/or cellular cholesterol on osteoblast cell proliferation, differentiation, and bone health. Findings of these studies are summarized in Table 1. In 1999, it was shown for the first time that the treatment of rodents with cholesterol-lowering drugs simvastatin and lovastatin both increased bone formation (10). They had found that intra-calvaria injection of these statins showed an increased calvarial bone formation, and similarly, oral administration increased cancellous bone formation (10). Later on, our research group had reported that treatment of osteoblast 2T3 cells with simvastatin and lovastatin increased alkaline phosphatase (ALP) activity (an early osteoblast differentiation marker), and expression of collagen and OPN (late osteoblast differentiation markers) (18). As a mechanism, it was found that simvastatin treatment increases the expression of the potent osteoinducer BMP-2, by upregulating Akt and Erk1/2 activity to enhance osteoblast differentiation. We and others have reported that statins upregulate BMP-2 expression to increase osteoblastic ALP activity and nodule formation (10, 18, 41, 42, 43). Similarly, simvastatin treatment was also found to increase cell proliferation and osteoblast differentiation of human periodontal ligament cells (PDLs) with concomitant increase of ALP, OPN, and mineralization (44). However, simvastatin was not found to influence proliferation/viability of PDLs (45). However, proliferation/viability of primary alveolar osteoblast cells (AOBs) was significantly decreased by the treatment of simvastatin at different concentrations (45). Simvastatin treatment (1 and 100 nM) was able to increase ALP activity of both AOBs and PDLs. Simvastatin and pravastatin treatments were found to increase the activity of ALP and nodule formation (46, 47). However, these treatments showed reduction of ALP and mineralization in aortic valve myofibroblast cells (46), whereas mevastatin failed to increase ALP activity and calcium incorporation into cells (48). Thus, the substantial data showed an increased osteoblastic differentiation, when osteoblast or osteoblast-like cells were treated with statins. But, the cell proliferative response upon statin treatment depends on cell types, type of statins, its dosage, and duration of exposure.

**Table 1:** **Effect of cholesterol or cholesterol reducing drugs on osteoblast activity**.

Cell types and animal models	Treatment	Dose	Duration	Regulated genes	Activity	Reference
Osteoblast MC3T3-E1	Cholesterol	12.5–50 μg/ml	72 h	Decreased BMP-2, ALP, Runx2, Collagen A1	Decreased proliferation	(41)

Mouse mesenchymal stem cells	Cholesterol	5–15 μg/ml	48 h	Increased ALP, BMP-2, Runx2, ALP, and OCN	Increased cell number and nodule formation (after 14 days of treatment)	(42)

Vascular smooth muscle cells	LDLR^−/−^	–	–	Decreased ALP (decreased intracellular cholesterol)	Decreased mineralization	(49)

M2-10B4 (mouse marrow stromal cells)	Cholesterol-derived products Oxysterol (20(S)-hydroxycholesterol 22(S)-hydroxycholesterol	5 μM	Exposure time 1–8 h	Increased ALP and OCN (after 4 days)	Increased Calcium incorporation (after 14 days) Increased bone volume	(50, 51)

MCF7 breast cancer cells	Osteoblast (MG63) derived oxysterol	Conditioned medium	24 h	–	Increased cancer cell migration	(52)

Rat (ovariectomized)	High cholesterol	3% Cholesterol	3 months	Increased IL6	BMD less (femur)	(41)

MG63 and 2T3	Simvastatin	2.5 μM	48 h	BMP-2 mRNA and protein		(10)

Calvaria bone (mice)	Simvastatin and lovastatin	0.062–0.25 μM	72 h	–	Increased bone area	(10)

Human periodontal ligament cells	Simvastatin	0.01–0.1 μM	24 h	Increased ALP and osteopontin (after 7 days). Decreased ALP (at 10^−7^M)	Increased cell proliferation, increased calcium content (after 21 days)	(44)

Human periodontal ligament cells	Simvastatin	0.001–0.1 μM	24–72 h	Increased ALP, OCN, OPG and RANKL	Not effect on proliferation and viability	(45)

Primary alveolar osteoblast cells	Simvastatin	0.001–0.1 μM	24–72 h	Increased ALP, OCN, OPG and RANKL	Decreased cell proliferation and viability	(45)

Osteoblast MC3T3-E1	Simvastatin	0.1 μM	4 h (treatment)	–	Increased H_2_O_2_-inhibited osteoblast viability, decreased apoptosis, and increased osteoblast differentiation	(53)

Osteoblast MC3T3-E1	Simvastatin + BMP-2	0.1–1.0 μM	5 days	Synergistic increased Psmad1/5 and ALP	Decreased cell growth	(54)

Mouse marrow stromal cells (M210B4)	Mevastatin	1.0–3.0 μM	2–8 days	Not effect on OCN, decreased ALP	Decreased calcium incorporation and cell number	(48)

Primary cultured marrow stromal cells, rat (adult)	Simvastatin	0.1–1.0 μM	10 days	Increased ALP and OCN	Increased mineralization (21 days), inhibited adipogenesis	(47)

Aortic valve myofibroblast cells	Simvastatin and pravastatin	0.1–0.6 μM	–	Decreased ALP	Decreased mineralization	(46)

M2-10B4 mouse marrow stromal cells	Simvastatin and pravastatin	0.1–0.6 μM	–	Increased ALP	Increased mineralization	(46)

2T3 osteoblast cells	Lovastatin	5.0–10.0 μM		Increased BMP-2, ALP, collagen, OPN	–	(18)

MC3T3-E1 osteoblast	Simvastatin, cerivastatin, atorvastatin	0.1 μM	4–20 days	Increased BMP-2 (6 days), ALP (12 days), type I collagen, BSP (20 days), and OCN (20 days)	Increased mineralization (24 days) (for all statins)	(55)

Mice (ovariectomized)	Simvastatin	1–10 mg/kg/day	35 days	–	Increased trabecular bone volume and decrease osteoclast number	(10)

Mice (ovariectomized)	Simvastatin	10 mg/kg/day	13 weeks	–	No change on trabecular bone volume	(56)

*ALP, alkaline phosphatase; OCN, osteocalcin; OPN, osteopontin; BSP, bone sialoprotein; RANKL, receptor activator for nuclear factor kappa B ligand; OPG, osteoprotegerin; BMP-2, bone morphogenetic protein-2*.

In animal studies, high cholesterol diet showed a reduction of BMD in femur with concomitant increase of serum OCN and carboxyterminal collagen crosslinks (CTX), which suggests that high cholesterol may increase bone turn over (41). Moreover, treatment of osteoblast MC3T3-E cells with different concentrations of cholesterol inhibited proliferation and differentiation (41). Cholesterol treatment reduced osteoblastic proteins Runx2, ALP, and collagen A1 with a concomitant decrease of BMP-2. These findings suggest that cholesterol may target BMP-2 to block the expression of Runx2, ALP, and collagen 1A in osteoblast cells, which in turn inhibits osteoblast differentiation. However, recent evidence showed that a high cholesterol diet was capable of masking the type 2 diabetes-induced bone loss (57). Other evidence documented that cholesterol-treated mesenchymal stem cells (MSCs) showed an increased expression of osteogenic lineage markers, increased ALP activity, and more mineralized nodule formation. It was noted that osteogenic potency of cholesterol was mostly due to elevation of cholesterol ester (42). These contrasting findings suggest that functional activity of cholesterol depends on disease condition, types of cholesterol (free cholesterol and cholesterol ester), and cell types, akin to statins.

Similarly, oxysterol, a cholesterol-derived product, showed induction of ALP, OCN, and mineralization in marrow stromal cells, and decreased adipocyte differentiation (50, 51). As a mechanism, it was found that oxysterol increased osteogenic markers by inhibiting DKK-1, a known Wnt inhibitor, but the blocking of PI3K pathway was shown to reverse the oxysterol-induced osteogenesis in these stromal cells (58). Moreover, oxysterol prevented oxidized LDL-inhibited osteoblast differentiation of marrow stromal cells (59). However, the primary cholesterol metabolite, 27 hydroxycholesterol, through its actions on both estrogen receptors and liver X receptors, decreased osteoblast differentiation and enhanced osteoclastogenesis, resulting in increased bone resorption (60).

Vascular smooth muscle cells isolated from LDL receptor (LDLR) knock-out mice showed reduction of ALP activity and matrix mineralization with a concomitant decrease of intracellular cholesterol when compared with wild type cells (49). Schilling et al. showed that apolipoprotein E (APOE) deficient mice showed an increased bone mass as compared to control mice (61).

These data indicate that different cholesterol products may have distinct roles in osteoblast- or osteoblast-like differentiation, but decreased of cellular cholesterol may prevent vascular calcification, whereas increased cholesterol may inhibit osteoblast differentiation to prevent bone formation.

## Cholesterol and Osteoclasts

Almost two decades ago, the Mundy research group suggested, based on rodent model experiments that statins might prevent osteoporosis by increasing osteoblastic differentiation and decreasing bone resorption (10). At that time, some clinical data supported the concept that cholesterol-lowering statin drugs might decrease the fracture risk of bone (62). Von Stechow et al. observations did not correlate with these findings (56). It was reported that treatment of macrophages with cholesterol showed an enhancement of osteoclast activity by increasing the expression of the interleukin alpha gene (63). Similarly, the removal of cellular cholesterol from osteoclast precursor cells by treating with cyclodextrin or high-density lipoprotein (HDL) was found to increase apoptosis of osteoclast cells (64). Depletion of cellular cholesterol has prevented RANKL- or CSF-1-driven osteoclast activity, presumably by inhibiting the cell survival Akt–mTOR–S6 pathway (64). Moreover, LDLR-deficient osteoclast cells that might have low cellular cholesterol showed reduced size and life span when compared to wild type cells. Similarly, blocking the mevalonate pathway inhibited osteoclast activity, and also prevented apoptosis of osteoblast (65). More evidence showed that the depletion of lipid raft-cholesterol inhibits H-RAS-induced expression of RANKL to mitigate osteoclast activity (66). Similarly, the integrity of lipid rafts regulate V-ATPase activity in osteoclast, indicating that the removal of cholesterol from lipid rafts may impair osteoclast function (67). All these data together suggest that the cellular cholesterol of osteoclast cells might positively influence the bone deterioration by enhancing osteoclast activity and preventing osteoclast apoptosis. However, cellular cholesterol may have a contrasting function in regulating apoptosis of osteoblast and osteoclast cells (64, 65).

Lovastatin treatment inhibited osteoclast differentiation by reducing of geranylgeranylation of prenylated proteins that inhibition of geranylation and farnesylation has been shown to be the mechanism of action of nitrogen containing bisphosphonates, which are used to inhibit osteoclastic bone resorption (68, 69). Moreover, atorvastatin treatment to primary osteoblast cells showed an increased level of OPG, decoy receptor of RANKL, which prevents osteoblast-aided osteoclast activity by blocking the binding activity of RANKL to RANK receptor of osteoclast cells (70). It was also reported that RANKL expression of AOBs was increased with the treatment of simvastatin at different concentrations (low to high), but this expression of PDLs was increased only when higher concentrations of simvastatin (10–100 nM) were used (45). These findings indicate that statins may have a different cellular response in regulating RANKL expression. In these studies, they did not check the effect of simvastatin on osteoclast activity, but the expression of OPG level was increased in both cell types in response to simvastatin treatment (45). Since earlier studies documented that statin treatments might also increase the OPG level to block RANKL function (70). Thus, the RANKL/OPG ratio upon statin treatment can only predict the osteoclast activity.

Animal-based studies further indicate that the mice with hypercholesterolemia reduce the formation of cortical and trabecular bone in the femurs and vertebrae by promoting osteoclastogenesis (71). Moreover, under hypercholesterolemia condition, mice showed a higher risk of fracture. Similarly, high fat diet also induces cathepsin K positive osteoclast cells and RANKL expression, leading to enhanced osteoclastogenesis (72). Interestingly, ovariectomized (OVX) mice exhibit enhanced bone resorption and bone loss due to deficiency of estrogen, but simvastatin-treated OVX mice showed a reduction of bone loss by decreasing osteoclast activity (73). Similarly, in a rat model, a high cholesterol diet caused an enhancement of bone resorption with a concomitant increase of TRAP positive osteoclast cells (74).

Thus, the substantial data of cellular, animal, and clinical research studies suggest that the reduction of cholesterol might prevent bone loss by inhibiting osteoclast activity. Results of these studies are summarized in Table 2.

**Table 2:** **Effect of cholesterol or cholesterol reducing drugs on osteoclast function**.

Types of cells, animal model, clinical data	Treatment for alteration cholesterol level	Regulated genes	Activity	Reference
Macrophages	Cholesterol	Increased IL6	Increased osteoclast activity	(63)
Osteoclast cells	Removal of cholesterol (by cyclodextrin or HDL)		Inhibited RANKL/CSF-1-induced osteoclast activity	(64)
Murine preosteoblast (CIMC4)	Removal of cholesterol (by cyclodextrin)	Inhibited RANKL	–	(66)
Bone marrow-derived osteoclast	Removal of cholesterol (by cyclodextrin)	Inhibited V-ATPase	Inhibited osteoclast activity	(67)
Human primary osteoblast cells	Atorvastatin	Increased OPG	Inhibited osteoclast activity	(70)
Breast cancer and multiple myeloma	Simvastatin	Inhibited RANKL l-induced NFκB pathway	Inhibited RANKL-induced osteoclast	(75)
Ovariectomized rat	Simvastatin	Decreased Trap	Decreased osteoclast	(73)
Rat	High cholesterol	Increased Trap	Increased osteoclast	(74)
Mice	High cholesterol		Increased osteoclast	(71)
Rabbit	High fat diet	Increased RANKL, MCP-1, Cathepsin K	Increased osteoclast	(72)
Multiple myeloma patients	Simvastatin	Increased Trap (serum)	Bone resorption	(76)

*RANKL, receptor activator for nuclear factor kappa B ligand; OPG, osteoprotegerin; CSF-1, colony stimulating factor-1; Trap, tartrate resistant acid phosphatase; MCP-1, monocyte chemoattractant protein-1*.

## Cholesterol and Bone Metastasis

**Figure 2:**
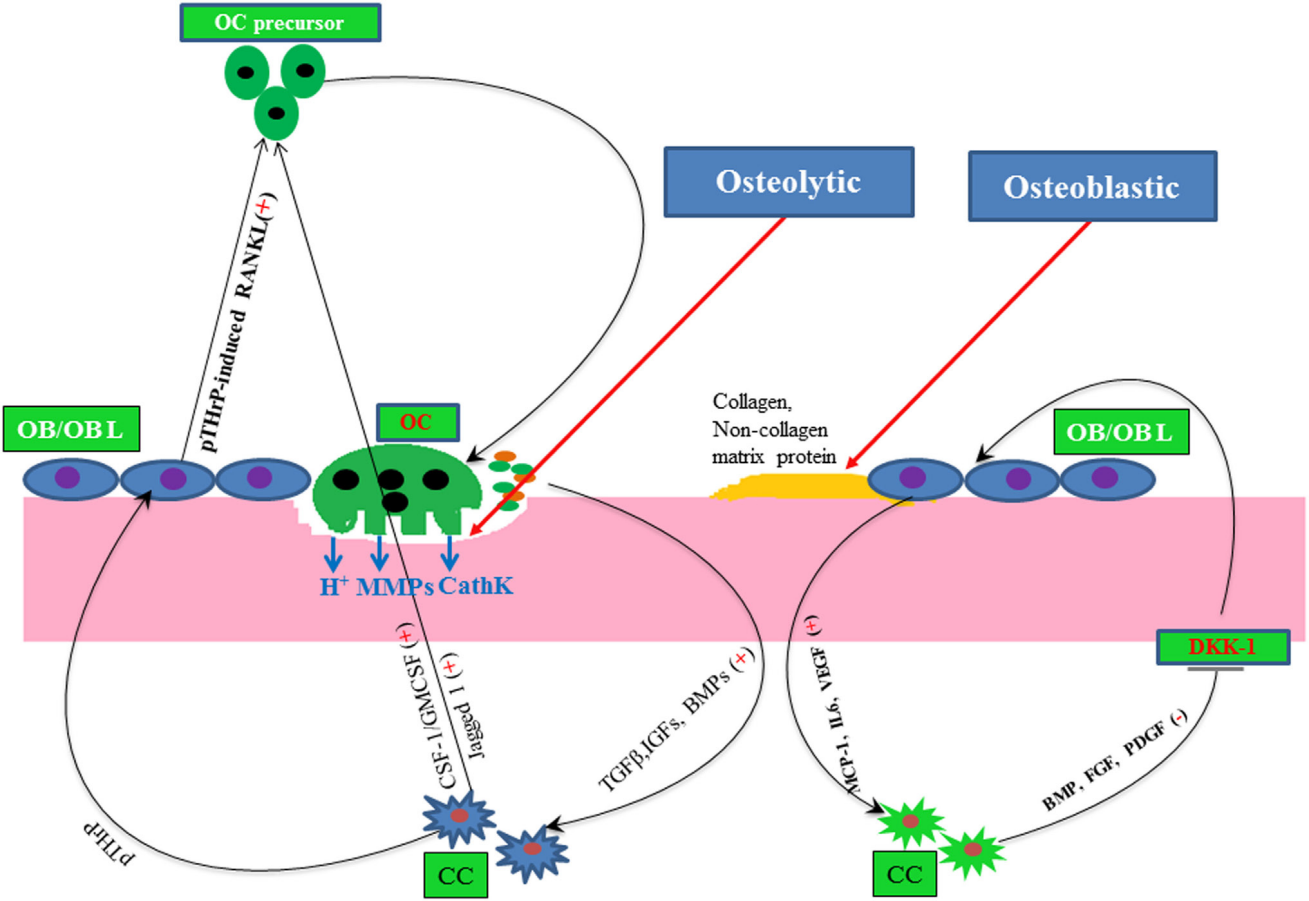
**A simple molecular view of osteolytic and osteoblastic metastasis of cancer**. Here, plus and minus signs depict positive and negative regulator, respectively, line arrow head and blunt ended line depict increased and decreased function, respectively. Abbreviations: OC, osteoclast; OB, osteoblast; OBL, osteoblast lineage; CC, cancer cells; CathK, cathepsin K.

Bone is one of the most common sites for the metastasis of many cancer types, such as breast, prostate, ovarian, and lungs, since bone provides a fertile soil to disseminated tumor cells (77). During bone metastasis, metastasized cancer cells interrupt the normal bone remodeling program either by increasing osteoclast activity or by altering the osteoblast activity, as depicted in Figure 2. For example, breast cancers mostly show osteolytic, and prostate cancers often undergo osteoblastic metastasis. But in both cases, either abnormal osteoclast or osteoblast activity leads to increase the risk of bone fracture. Many growth factors and cytokines have been shown to participate in regulating bone metastasis. For example, osteoclastogenic CSF-1 plays a pivotal role in bone remodeling, but the abnormal expression of this cytokine correlates with bone metastasis and osteoporosis (78–81).

It has been demonstrated that hyperlipidemia and high cholesterol promote cell migration and metastasis (13, 15). The detailed molecular mechanism is not yet known as to how high cholesterol increases tumorigenesis. Although, it was hypothesized that statins may prevent skeletal metastasis of cancers (82). Our study, for the first time, experimentally documented that simvastatin treatment inhibits osteolytic bone metastasis, which was developed after intracardiac inoculation of human metastatic breast cancer MDA-MB-231 cells in mice (19).

It was suggested that simvastatin treatment increased tumor suppressor protein p53 levels to transcriptionally repress breast cancer stem cell marker CD44 expression. This in turn inhibits the breast cancer cell migration and invasion, resulting in the inhibition of osteolytic metastasis (19). Later on, another research group had demonstrated that simvastatin treatment prevents osteolytic lesions of the spinal cord, which was produced due to injection of the lung cancer A549 into nude mice. Similar to our observation, they had also suggested that simvastatin treatment upregulated p53 protein with concomitant increase of CD44, MMP-2, and MMP-9 (83). Our research study also proved that metastatic breast cancer cells secrete more CSF-1 as compared to non-metastatic breast cancer cells (84). Other study reported that statins inhibit CSF-1 expression (85). These data suggest that breast cancer cell-derived CSF-1 might potentiate osteolytic bone loss, which could be inhibited by the treatment of statins. Recently, a study has shown that the combination of simvastatin and the anti-diabetic drug metformin synergistically reduced the metastatic property of cancer cells as well as metastasis to the spinal cord and femurs. In this study, prostate cancer C4-2B cells were injected into castrated aythmic mice to develop metastasis (86). The combination of these two drugs inhibited AKT and AMPKα activity (86). We had reported earlier that simvastatin treatment upregulated the tumor suppressor protein PTEN. PTEN inhibits AKT activity in preventing cancer growth both in *in vitro* and an animal model (87). These findings indicate that cholesterol may positively modulate bone metastasis of cancers. Clinical evidence supports this concept, since the mean cholesterol level was found to be increased in bone metastases when compared to healthy controls (88).

**Figure 3:**
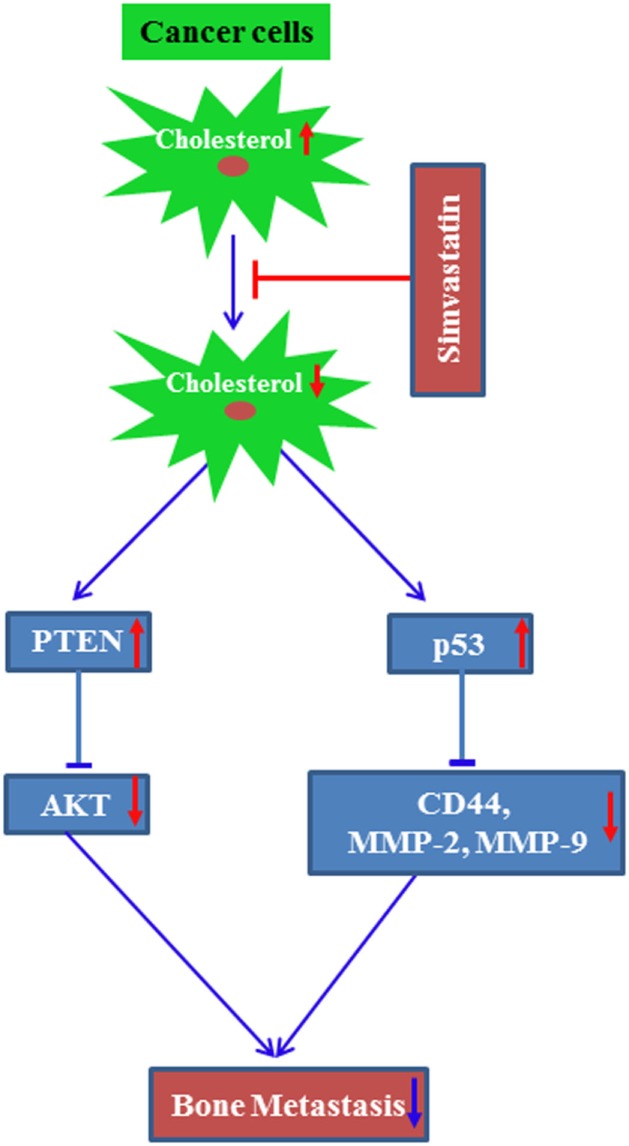
**A proposed molecular mechanism by which simvastatin inhibits bone metastasis of cancer**. Line arrow head and blunt ended line depict increased and decreased function, respectively, and up and bottom arrow show increased and decreased level/expression, respectively.

Altogether, these data support the idea that cellular cholesterol might potentiate bone metastasis of cancers. Based on these findings, a potential molecular mechanism by which statins inhibit cholesterol-mediated bone metastasis has been proposed (Figure 3).

## Cholesterol and Multiple Myeloma

Clinical findings suggest that serum cholesterol level of myeloma patients is low, since myeloma cancer cells consume more serum cholesterol for their growth (89). Exogenous treatment of cholesterol increases cell survival of myeloma cells (90). Myeloma cancer cells may increase cellular cholesterol either by inhibiting cholesterol efflux or increasing sterol regulatory element binding (SERB) transcription factors, which transcriptionally increase HMGCOA reductase and LDLR expression (91). More evidence showed that simvastatin treatment was quite capable to inhibit myeloma driven osteoclast activity, which was promoted by RANKL treatment (75). In contrast to the above studies, Sondergaard et al. reported that a high dose of simvastatin treatment to multiple myeloma patients showed elevated levels of TRAP and CTX in serum, indicating that simvastatin treatment might increase bone resorption, instead of inhibition (76).

These conflicting results indicate that cholesterol-lowering drugs may prevent myeloma-induced bone diseases, provided the drugs reduce the cellular cholesterol of the myeloma cells.

## Conclusion

Low BMD and the occurrence of bone fractures are strong predictors for osteoporosis. Many research studies have strengthened the link between the serum lipid profile and BMD. Here, the relationship between cholesterol and BMD has been summarized in Table 3. Substantial data showed a negative association between BMD and serum TC, and LDL cholesterol (5, 7, 8, 92–97). A few studies found no association and/or a positive association with serum cholesterol and LDL cholesterol (98–101). The summarized data from all the studies indicate that there is an existence of an inconsistent relationship for the case of HDL cholesterol and BMD (6, 99, 100, 102). In several clinical trials, but not in all, the use of statins have been associated with a reduction of fracture risk, and the patients taking statins having a higher BMD than those who do not (103–105). In brief, a high cholesterol level is inversely associated with BMD, and statin treatment shows an enhancement of BMD, indicating high cholesterol as a negative regulator of bone health.

**Table 3:** **Relationship between cholesterol and BMD**.

Lipid types	Relationship between BMD and cholesterol	Subjects	Reference
LDL cholesterol	Inverse association with BMD at 1/3 radial, distal radial, and lumbar	Postmenopausal women	(92)
Cholesterol	Inverse association with BMD at lumbar spine and distal forearm but not with hip	Postmenopausal women	(94)
Cholesterol and LDL cholesterol	Inverse association with BMD at spine and hip	Postmenopausal women	(93)
Cholesterol and LDL cholesterol	Inverse association with BMD at spine, hip, and forearm	Postmenopausal women	(96)
Cholesterol and LDL cholesterol	Inverse association with BMD at lumbar and femoral neck	Early postmenopausal women	(95)
Cholesterol and LDL-cholesterol	Inverse association with BMD at trochanter, shaft and proximal total hip	Pre and postmenopausal women	(97)
Cholesterol and LDL-cholesterol	Inverse association with BMD at lumbar and whole body	Postmenopausal women	(5)
Cholesterol	Inverse association with BMD	Premenopausal women	(8)
Cholesterol/HDL cholesterol and LDL cholesterol/HDL cholesterol	Inverse association with BMD	Men with dyslipidemia	(7)
Cholesterol and LDL cholesterol	No association with BMD at lumbar spine and femur neck	Postmenopausal women	(99)
Cholesterol and LDL-cholesterol	No association with BMD at femoral neck, trochanter, intertrochanteric zone, and lumbar vertebrae	Male	(100)
Cholesterol and LDL cholesterol	No association with BMD	Premenopausal women	(101)
Cholesterol	Positive association with BMD at total body and at all sites but not with neck	Postmenopausal women	(98)
HDL cholesterol	Positive association with BMD at 1/3 radial, distal radial, and lumbar	Postmenopausal women	(92)
HDL cholesterol	Positive association with BMD at trochanter	Postmenopausal women	(6)
HDL cholesterol	Positive association with BMD at femur neck	Postmenopausal women	(99)
HDL cholesterol	Positive association with BMD at femur neck	Male	(100)
HDL cholesterol	Inverse association with BMD at femur neck and total hip	Premenopausal women	(101)
HDL cholesterol	Inverse association with BMD at femur neck	Pre and postmenopausal women	(102)

*LDL, low-density lipoprotein; HDL, high-density lipoprotein; BMD, bone mineral density*.

However, the existence of this inverse relationship was not always found (Table 3). Thus, it could be that serum cholesterol or cholesterol-lowering drugs might have a variable influence in regulating BMD at different sites. The effect of serum cholesterol on BMD may vary among individuals, by gender, with age, and menopausal status of women. However, if we summarize all results of cell culture and animal experiments (Tables 1 and 2), it can be concluded that high cholesterol often increases osteoclast activity and decreases osteoblast function, especially in bone residential cells. It can also be concluded that targeting cellular cholesterol might decrease osteoclastogenesis with an enhancement of osteoblast function of bone cells (Tables 1 and 2). However, further research indicates that osteoblast activity in response to statin treatment markedly depends on cell types, dosage, and type of statins, but in most cases, statin treatment has been found to inhibit osteoclast or bone resorption activity (Tables 1 and 2). In cancers, most of the literature suggests that instead of serum cholesterol, cellular cholesterol might influence the cancer progression and metastasis (16, 38). Moreover, bone metastases contained an elevated level of cholesterol when compared to normal tissues (88). Similarly, depletion of cellular cholesterol prevents apoptosis of osteoblast cells, and increases apoptosis of osteoclast cells (64, 65).

Therefore, cellular cholesterol levels of bone occupant cells (such as osteoblast, osteoclast, osteocyte, myeloma, and bone metastasized cancer cells) might be a good predictor of the patient’s bone health, and hence, targeting the intracellular cholesterol might improve bone health.

## Conflict of Interest Statement

The author declares that the research was conducted in the absence of any commercial or financial relationships that could be construed as a potential conflict of interest.
